# Supplementary material for: Crawling and Gliding: A Computational Model for Shape-Driven Cell Migration
Source: PLoS Comput Biol. 2015 Oct 21;11(10):e1004280. doi: 10.1371/journal.pcbi.1004280 (PMC4619082; doi:10.1371/journal.pcbi.1004280)
Supplement: S1 Code — (ZIP) [file pcbi.1004280.s012.zip › release/tst/doc/html/classInfo-members.html]

Tissue Simulation Toolkit: Member List


Tissue Simulation Toolkit
 0.1.4.1


- Main Page
- Namespaces
- Classes
- Files

- Class List
- Class Hierarchy
- Class Members

Info Member List

This is the complete list of members for Info, including all inherited members.

|  |  |  |
| --- | --- | --- |
| ClickCell(Graphics \*g) | Info |  |
| Info(Dish &dish, Graphics &graphics, std::ostream &out=std::cout) | Info |  |
| Menu(void) | Info |  |
| WriteCOM(int cell\_id, std::ostream &out=std::cout) | Info |  |


---

Generated on Thu Aug 14 2014 22:04:01 for Tissue Simulation Toolkit by  

 1.8.6
